# Precious Essences: Female Secretions Promote Sperm Storage in *Drosophila*

**DOI:** 10.1371/journal.pbio.1001191

**Published:** 2011-11-08

**Authors:** Mariana F. Wolfner

**Affiliations:** Department of Molecular Biology and Genetics, Cornell University, Ithaca, New York, United States of America

## Abstract

Sperm that females receive during mating are stored in special places in the females' reproductive tracts. These storage sites serve to support and retain the sperm, maintaining the sperms' motility and, in mammals, permitting final sperm-maturation. The molecules that attract sperm to these sites and mediate what happens to them there have remained elusive. New research, using elegant genetic tools in *Drosophila*, shows that secretory cells associated with a sperm storage organ are important in sperm-supportive functions. When females lack function of these cells, they do not store sperm, or the sperm that they do store lose motility. Intriguingly, these effects influence gametes beyond the secretory cells' immediate vicinity. Loss of these cells eliminates the motility of sperm stored elsewhere in the reproductive tract and prevents the movement of eggs through the tract to exit the female. As a result of the latter problem, fertilized eggs hatch inside female flies that lack these secretory cells: instead of laying eggs, these females can “give birth” to live offspring. Because the cellular source of these gamete-regulating substances is now known, future studies can identify the specific molecules and mechanisms by which a female attracts sperm into storage and regulates the movement of sperm and eggs within her reproductive tract. It will be fascinating to determine how these molecules and mechanisms maintain gametes in active and viable forms and how evolution can modulate this to result in diverse reproductive strategies. Identification of these molecules also has potential practical implications for strategies to regulate the reproduction of insects of medical or agricultural importance.

## Mated Females Store Sperm

Long after mating, a female retains a special memento of the experience: within her reproductive tract, her mate's sperm continue to live and move. Sperm can remain alive and active in the female for quite some time, from a matter of days after mating in most mammals (though for months in hibernating little brown bats) to weeks, months, or even years in some reptiles and many insects (for review, see [1]–[3]). The record-holders are ants and bees: sperm from a single mating flight can remain viable and functional within the queen for decades (reviewed in [4]).

The prolonged presence of sperm in females can have several consequences for those females and their mates. It allows the production of progeny for a long time, even after only a single mating or a short-term bout of matings, in several taxa. This can be important in species in which males are hard to find, and can “carry” potentially useful male-derived gene variants through periods in which they might have been disadvantageous in whole animals [5]. When sperm from more than one male reside within the female, a common phenomenon in the animal kingdom, the female environment can bias which male's sperm get a better chance to fertilize her eggs [6]. Conversely, there can also be competition between the sperm from different males [7] so that the “best” male wins. Long-term presence of sperm in a female can affect her physiology or behavior in cases (such as fruit flies) where the sperm carry (and gradually release) molecules that trigger post-mating responses [8]. And, within the female, sperm can acquire motility or other traits that allow them to successfully fertilize an egg. For example, mammalian sperm are not capable of fertilization when first released from the male. Within the female's reproductive tract, the sperms' proteins, membranes, and motility are modified, making them capable of fertilizing an egg (for review, see [3],[9],[10]). This process of “capacitation” can be mimicked in vitro by buffers that contain an appropriate balance of critical ions and other factors, but the molecular means by which it occurs within the female reproductive tract is not fully understood. Similarly, honeybee sperm can be kept alive in vitro for extended periods in a solution of known composition [11], but how that relates to what supports them in their natural environment of the female reproductive tract is unknown.

## Females' Sperm-Storage Sites Keep Sperm Viable and Active

What allows sperm to live so long within females? In some senses, stored sperm are not very demanding cells; they just need to stay alive and swim (and, eventually, find an egg and fertilize it). But in another sense, as stripped-down cells with no gene expression, they need a lot of help to survive. Seminal fluid, transferred along with sperm in the ejaculate, contains proteins and sugars that can support or nourish the sperm, or assist them in reaching or staying in storage in the female. But most seminal components do not persist in mated females for anywhere near as long as the sperm are stored (e.g., [12] for *Drosophila*). Something else has to support those sperm. That “something else” has to come from the female.

A clue to what the female provides in this inter-organism cellular interaction comes from following the sperm after mating. Sperm go to specialized sites within the female where they are stored. For example, in many mammals, such as mice and cows, sperm congregate in the spaces between mucosal folds in the oviduct, adhering to the epithelial wall (the oviductal sperm reservoir) [3]. In insects, sperm enter specialized storage organs (spermathecae and, in some cases, seminal receptacles). Sperm at their storage sites can be quite active, swimming around and, in mammals, acquiring hyperactivity [10] as they dislodge from storage to move up the oviduct to the egg.

It is reasonable, therefore, to imagine that the storage sites might provide molecules and an environment that maintains and regulates sperm and protects them from less supportive conditions elsewhere in the reproductive tract. In mammals, for example, sperm in the oviductal sperm reservoir are supported at these sites as they undergo capacitation [3]. Not all sperm are released from the storage sites at once, thus ensuring a continual supply of mature capacitated sperm for fertilization. In some insects, in vitro studies suggest that secretions from the storage sites can potentially counter harmful effects of environment or of secretions from rival males. In bees and ants, where queens mate with multiple males during their single mating flight, this mating frenzy engenders fierce sperm competition between the males. In vitro experiments suggest that some factors important in this competition are in the seminal fluid; sperm of a male bee or ant survive much better in his own seminal fluid than in the seminal plasma of a competitor. But if sperm from that mating flight are ultimately going to be stored for years within the queen, the in-fighting between ejaculates of rival males has to stop. denBoer et al. showed that female spermathecal secretions play this peace-making role, allowing sperm to survive and countering the negative actions of seminal proteins [13]. But what is the cellular source of the molecules that keep sperm “happy” once they are stored? And what attracts them into storage in the first place? A new study in *PLoS Biology* [14] begins to answer these questions, using *Drosophila*.

## Drosophila Sperm Storage

**Figure 1:**
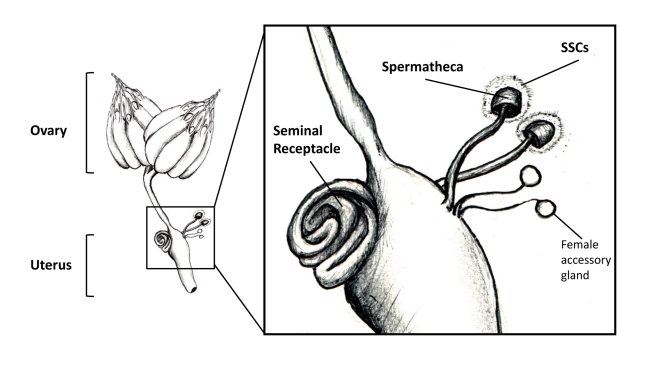
*Drosophila melanogaster* female reproductive tract. Mature oocytes leave the ovary and move through the oviducts to reach the uterus, where they can be fertilized prior to being laid. Sperm are stored in specialized organs (spermathecae, seminal receptacle; see inset for higher magnification) that open into the uterus near the site where fertilization will occur. Activity of the spermathecal secretory cells (SSCs) plays important roles in gamete dynamics within the female [14]. The inset also shows the female accessory glands (also called parovaria), which are believed to have a secretory function but are not sites of sperm storage. Drawing by J. L. Sitnik.

Mated *Drosophila* females store sperm in two types of organ: a long tube called the seminal receptacle and two spermathecae, which look like mushrooms [15] (see Figure 1). Stored sperm swim actively in both types of organ, including round and round in the spermathecae's donut-shaped caps [16]. Transcriptomic studies have identified molecules produced in each type of storage organ [17]–[21]. The organs' transcriptomes differ, suggesting that each organ has a unique function. This idea is supported by classical studies that suggest that the time of sperm entry into storage organs, and the length of time that they are retained in storage, differs between storage-organ types [22]. Studies using fluorescent sperm suggest that sperm from the seminal receptacle are most likely to fertilize an egg [16]. Although it was known that the female plays a role in getting sperm stored, through her nervous system [23] and via muscle contractions [24],[25], the specific cellular source of the molecules that attract sperm into storage, or support them there, was unknown. There was a good candidate, though; insect spermathecae are associated with secretory cells, and these cells seemed a likely source of molecules important for sperm storage. Sperm appear to have an affinity for these cells' secretions; within the spermathecae sperm are often seen associated with the secretions [26], and in *Anastrepha* fruit flies, some sperm heads even enter the ductules of the spermathecal glands [27]. Moreover, *Drosophila* mutants that lack spermathecae, or females whose spermathecae have been damaged, have problems in overall sperm storage [20],[28],[29].

## Spermathecal Secretory Cells Regulate Sperm—And Egg—Fates

A new study by Schnakenberg et al. shows that the spermathecal secretory cells (SSCs) are indeed the source of molecules that help get sperm into storage and that support the motility of stored sperm [14]. These authors used an elegant genetic method to study the SSCs' roles in vivo and to show that these cells' secretions act at specific times after mating. Intriguingly, the authors find that SSCs' effects are not only local within the spermathecae, but also long-range: SSC function is needed for proper sperm motility in the seminal receptacle. And, SSC function is needed to move eggs through the female's reproductive tract. Without SSC function, fertilized eggs can be retained for so long inside a female that they hatch there! This is the first report of ovovivipary in this species.

To manipulate SSC function, Schnakenberg et al. took advantage of transcriptome data that had identified genes that were expressed specifically in SSCs [21],[30],[31]. The authors used the promoters of two of these genes to drive expression of a cell death–inducing gene [32], thus killing the SSCs (and no other cells). One of the promoters they defined and used was expressed constitutively in SSCs; the other turned on in SSCs only after the female had mated. So Schnakenberg et al. could use the two promoters differentially: to kill SSCs in females at all times, or only after mating. By following the behavior of fluorescently labeled sperm, the authors showed that if females lacked SSC function, sperm would not enter their spermathecae. Intriguingly, if sperm entered spermathecae properly, but SSC function was then extinguished, the sperm remained in storage. Thus, something produced by SSCs attracts sperm into spermathecae and/or assists them in entering storage, but is not necessary to retain them there. The authors showed that SSCs did, however, affect an important behavior of stored sperm. In females that lacked SSCs, sperm that were stored in the other storage organ—the seminal receptacle—had impaired motility. This finding suggests that there can be communication between the sperm storage sites and explains a surprising result that had been reported previously: that ablation of spermathecae impaired all sperm storage, not just spermathecal sperm storage [20],[28].

## Perspectives

Schnakenberg et al.'s results get us closer to understanding how sperm are stored and maintained in mated females. And now that we know that the SSCs are the source of something that brings sperm into storage, we know to look among SSC secretions to discover what that “something” is. A logical next question would be how this molecule (or molecules) acts, and how sperm respond to it. But there are many other interesting questions to answer. For example, if SSCs produce a peptide or protein that attracts sperm, is its sequence highly variable within or between species, potentially contributing to sperm precedence by the female? It would also be fascinating to determine whether the female can modulate the amount of this attractant depending on how much sperm she already has in storage or on the attributes of the male with whom she is mating, given that males can adjust their reproductive secretions depending on their mates' characteristics [33]. Another mystery is how SSC-derived molecules act to influence the movement of sperm and eggs elsewhere in the female's reproductive tract. Such action could conceivably be mediated by something as simple as SSC secretions leaking out of the spermathecae, or by neural-based communications between the organs, but it is tempting to imagine that sperm, which have been seen to move between spermathecae and seminal receptacle [16], could themselves carry SSC secretions between the organs. Or might the spermatheca act like a spa, with sperm entering it to get “refreshed”? Restored to optimal speed and condition, perhaps even “rejuvenated”, sperm could then pop back into the seminal receptacle to rejoin the race to the egg. Given the identification of the cellular source of the molecules that lure sperm into storage and keep them active, we can now look forward to learning answers to these and other intriguing questions about the mechanisms by which females can influence sperm fate.

There are potential practical applications to extensions of Schakenberg et al.'s findings. For example, enhancing the reproduction of beneficial insects like honeybees, or interfering with the reproduction of harmful insects such as mosquitoes that transmit diseases, is potentially useful. Since insect fertility is reliant upon, or at least promoted by, storage of sperm within mated females, the molecules that regulate the storage of sperm, or the movement of eggs or sperm within females, provide “handles” for such manipulation. For example, a recent study of *Anopheles gambiae*, the mosquito that is the main vector of malaria, showed that disruption of a male-provided enzyme that is needed for storage of sperm by females impairs fertility [34]. The results of Schnakenberg et al.'s study of *Drosophila* suggest that disruption of SSC products in females would be valuable to explore as an alternative way to disrupt sperm storage in mosquitoes.

Schnakenberg et al.'s results also make an important contribution to the understanding of how reproductive strategies evolve. Although ovovivipary (eggs hatching within the female, resulting in live birth) has been reported in at least two other species of *Drosophila* [35], it not been reported before in *D. melanogaster*. Schnakenberg et al.'s results show that the *D. melanogaster* uterus is nevertheless capable of providing a hospitable environment for development through this stage. The authors point out that this finding suggests that the seemingly dramatic differences between ovipary (laying eggs that hatch outside the female) and ovovivipary may be simpler than anticipated. (Indeed, although there could be numerous reasons for ovovivipary in SSC-ablated females, it could even be as simple as the consequence of the absence of secretions that normally help to move eggs through the reproductive tract: eggs thus retained too long in those females hatch inside the uterus, and the larvae are supported there.) Schnakenberg et al.'s finding that only a few changes may underlie the distinction between two seemingly disparate reproductive strategies is a fascinating new contribution to the consideration of the evolution of life history traits.

## Abbreviations

SSC: spermathecal secretory cell
